# Sydenham Society

**Published:** 1850-03

**Authors:** 


### THE MEDICAL EXAMINER.

PHILADELPHIA, MARCH, 1850.

### SYDENHAM SOCIETY.

By the late report of this Society, we learn that there have been issued during the year ending May 1st, 1849, four volumes, viz., *Rhazes on the Small Pox and Measles; the first volume of the complete works of Hippocrates; the first volume of the English edition of Sydenham, and the second volume of Rokitansky's Pathological Anatomy.* The council have also secured the valuable services of Dr. Fleetwood Churchill, (well known to the profession in this country as the author of a treatise on Midwifery; a work on the diseases of Women; and one on diseases of Children,) to prepare a volume of select monographs, principally by English authors, on the diseases peculiar to women. This has been already issued in England, and contains much valuable matter on the subject of puerperal fever; the whole work indeed, with the exception of about fifty pages, being occupied with this subject. Dr. Sharpey, it is also announced, will edit a volume that will comprise the works of some of the most distinguished physiological writers of the age of Sydenham. They likewise announce that they will publish another selection from the clinical lectures of Dupuytren, on the diseases and injuries of the bones, and one volume of the British Medical Bibliography, already announced.

Our object in bringing this subject before our readers, is to direct attention to the advantages gained by membership. The great aim in the institution of this Society, was to meet certain acknowledged deficiences in existing means for diffusing medical literature, which were not likely to be supplied by the efforts of individuals. To carry this object into effect, they propose a succession of publications, embracing, among others, reprints of standard English works, which are rare and expensive; miscellaneous selections from the ancient and from the earlier modern authors, reprinted or translated; digests of the works of old and voluminous authors, British and foreign, with occasional biographical and bibliographical notices; translations of the Greek and Latin medical authors, and of works in the Arabic and other Eastern tongues, accompanied, when it is thought desirable, by the original text; translations of recent foreign works of merit; original works of merit which might prove valuable as books of reference, but which would not otherwise be published, from the slender chance of their meeting with a remunerating sale—such as Bibliographies, alphabetical and digested Indexes to voluminous periodical publications, &c.

These works are handsomely printed and bound on a uniform plan, and distributed to the members, subject, however, to a due share of the carriage of the parcel in which the books are sent. The price of subscription is five dollars, to be paid in advance, for which every member is entitled to a copy of each work for the year for which he subscribes. The price of the subscription may be forwarded to either of the local secretaries, Dr. R. Dunglison, Philadelphia, or to Dr. Lee, New York.

Those who are desirous of becoming members can do so by transmitting their names and address, with the amount of subscription, to either of the local secretaries above named.

No one who examines into this matter, can fail to be struck with the advantages resulting from membership, in the value of the publications that have already been presented to the members, and the low rate at which they are supplied. In the last two years, eight large and handsomely printed volumes have been distributed, and the announcement for the present year gives evidence of still greater liberality on the part of the council. We trust that their efforts to disseminate sound medical literature among the profession may meet with the success it deserves.

---

Under the department of Surgery, in the Record of the present number, will be found a report of a very ingenious and successful operation for the relief of deformity from a burn, by Dr. S. P. Hullihen, of Wheeling, Va. The report was sent to us in pamphlet form, and we have been induced to present it to our readers, under the conviction that they will form as high an estimate of the judgment and surgical skill of Dr. H. as we have been led to do from its careful perusal. We have also read with much pleasure an essay on cleft palate and its treatment, and one on hare lip and its treatment, from the same source.

